# Community health workers: a narrative review of a curriculum and training program for low-income communities facing limited access to healthcare

**DOI:** 10.3389/fpubh.2025.1504490

**Published:** 2025-04-11

**Authors:** Maryam Othman, Gary W. Selnow

**Affiliations:** ^1^Department of Population Health Science, College of Osteopathic Medicine of the Pacific, Western University of Health Sciences, Pomona, CA, United States; ^2^Independent Researcher, San Francisco, CA, United States

**Keywords:** cost-free community health worker training, low-resource community health preparedness, closing healthcare equity gap, online and downloadable free CHW training curriculum, local health worker education, free CHW continuing medical education program

## Abstract

**Background:**

The aim of this narrative review is to examine a WHO-compliant program that prepares Community Health Workers (CHWs) in low-resource environments. The intended outcome of the training is to enhance healthcare access and address health equity disparities. We examined the program’s curriculum, instructional methods, and a complementary continuing medical education (CME) program designed to sustain CHWs’ knowledge and skills.

**Methodology:**

We review, in detail, the CHW training program—its curriculum, delivery, and implementation—launched prior to the COVID-19 pandemic and continuing today. This program develops critical human resources to expand the reach of overburdened healthcare professionals in disproportionately affected regions.

**Results:**

Our review highlights the positive impact of this program on marginalized communities. We propose the adoption of its curriculum and pedagogical framework by local leaders seeking to train teams of well-prepared CHWs to improve healthcare and bridge the gap between communities and medical professionals. This no-cost program is accessible even to the most under-resourced settings.

**Conclusion:**

The program examined in this paper offers small, disproportionately affected communities a valuable opportunity to implement a program that equips CHWs to provide essential clinical services and conduct community-wide health education initiatives. These CHWs serve as vital bridges between their communities and the professional medical system. Their demonstrated effectiveness in serving the health needs of their communities, even during the challenging COVID-19 pandemic, is noteworthy. When called for, they can support refugees fleeing war and climate pressures by offering basic clinical care and prevention training.

## Introduction

The World Health Organization (WHO) for years has reported its concerns about the shortage of medical professionals worldwide and particularly in low-resource regions (1). This issue becomes only more pressing as the WHO and other agencies project a deficit of up to 18 million health workers globally by 2030 (2). More troubling, the burden of this shortage will continue to fall disproportionately on low-income countries, exacerbating the existing gaps in equitable access to healthcare worldwide (3). This disparity will become even more of a threat as illnesses related to climate change increase in the years ahead.

No one expects a sudden surge in the number of doctors and nurses to fill the gap. The most achievable solution to address health equity challenges, we believe, is an expansion of community health worker (CHW) teams throughout low-resource countries (4, 5). Recognizing their critical role as part of the healthcare mosaic, the WHO has long advocated for CHW involvement in basic care and illness prevention (6).

Community Health Workers have a well-established history of providing essential health services in regions with limited access to professional healthcare resources (7). Known by various names, such as traditional healers, *promotoras*, and village health promoters, these early health advocates have leveraged their deep understanding of local communities, and their unique skill sets to deliver a range of vital services (8, 9). With the right preparation, management, and ongoing training, CHWs can make a substantial contribution to improving healthcare access (10) and addressing the inequities seen ahead for low-resource environments (11).

The time and cost efficiencies of training and deploying CHWs can allow disproportionately affected communities to benefit from health services otherwise beyond their reach. Communities can prepare CHWs quickly and affordably, and within months establish operational teams that can extend the reach of professional healthcare providers and become bridges to official health systems (12).

The WHO provides guidelines to enhance the design, implementation, and performance of CHW programs (13). Several well-funded organizations actively promote CHW training and collaborate with governments and other institutions to facilitate that training. Their aims and efforts are commendable; however, they may not be appropriate for or available to small communities in low-resource regions. Consequently, distant, often overlooked populations, with limited means, would benefit from CHW training strategies that enable small communities to prepare CHWs without significant intervention and outside support (14).

We have identified a program constructed by WiRED International, a small non-governmental organization, that developed and deployed a WHO-compliant CHW program designed specifically for under-resourced environments. This evidence-based, professionally constructed program adopts a rigorous curriculum that is implemented at the local level by local leadership including small government groups, clinics, and non-governmental organizations (NGOs). In other words, the cost-free program offers professional-level CHW training, initiated and conducted by local leaders. Such an approach is not in competition with larger training programs but offers an alternative model for smaller communities. It allows for greater flexibility, community ownership, and responsiveness to local needs, which can be crucial for the success and sustainability of CHW initiatives.

## Pedagogical frameworks and learning environment

This CHW training program was developed from the ground up by a team of medical and health experts. Several principles guided the application and development of the pedagogy for this program:

- All elements of the program must be WHO-compliant.
- Training material must be professionally produced, evidence-based and peer reviewed.
- The curriculum (developed by medical experts) is to be presented in interactive training modules, available online and for download to smart phones and tablets. After downloading, the entire program should operate offline (given the limited connectivity in most target communities).
- The curriculum must use an active approach to learning, first through classroom instruction, then through ongoing student collaborations to reinforce skills development.
- The curriculum will be germane to populations globally but adapted to local needs as the WHO urges.
- Two strategies localize the program: (1) In-person classes are instructed by medical professionals from the same region to be served by the CHWs, (2) after core training, CHWs will participate in a cost-free, continuing medical education (CME) program.
- All material and services provided by the organization will be offered cost-free.

## Program details

### The curriculum

Over the course of 2 years, a panel of 12 medical professionals, including physicians, nurses, and medical professors, developed the curriculum. They carefully studied the World Health Organization’s (WHO) guidelines for CHW training and duties, as well as the related skill sets, and knowledge base required to carry out CHW duties. Along with their own extensive medical experience in low-resource regions, team members carefully assembled a comprehensive list of course topics—a detailed syllabus—for the training program. The writing team recognized the dynamic nature of the curriculum (15), expecting updates as needed to reflect scientific advances and changing health needs of communities. For instance, with the guidance of first responders, doctors and nurses, the team added a significant first aid and critical care component preparing CHWs to address minor emergencies where higher-level care is not immediately available (Table 1).

**Table 1:** Program details.

The curriculum	A panel of 12 medical professionals developed the evidence-based, peer-reviewed curriculum.
Topics	The curriculum comprises 34 modules, divided into four blocks: Core health, Clinical issues, First Aid and Critical Care, and Communication (including health surveillance).
Structure	Each module employs the principles of effective Cognitive Information Processing Theory.
Distribution	The CHW training program is distributed in two ways: through an internet website for online study; and through a mobile phone app for offline study in locations with intermittent connectivity.
The learning environment	The curriculum is presented in a 5-week classroom setting led by two local medical professionals.
Continuing medical education (CME)	A free online CME program with over 400 health and medical training modules enables CHWs to retain their knowledge and develop new skills.

### The topics

The curriculum comprises 34 modules, divided into four blocks: Core health, Clinical issues, First Aid and Critical Care, and Communication, which includes teaching methods and health surveillance strategies.

### The structure

Simple template structures the contents of each module. This helps learners navigate the material and know what to expect. Moreover, each lesson employs the principles of effective Cognitive Information Processing Theory, employing a repetition schedule that helps students transfer information from short-term to long-term memory (16).

At the end of each module, learners take an interactive, scored final exam, evaluating their overall comprehension and retention. The final exam provides feedback on each question, thus furthering a deeper learning of the material and reinforcing the key concepts.

### Distribution

Distribution of the CHW training program capitalizes on the growing adoption of mobile technology (17, 18). CHWs can access the interactive training modules online or download them to smartphones and tablets. This open-access digital distribution has several important benefits:

- **Immediacy**: Online distribution allows faster and more widespread adoption of the program.
- **Local initiative**: Local sponsors are able to evaluate and adopt the curriculum without administrative hurdles, upfront costs, or applications.
- **Accessibility**: Even in areas with limited connectivity, CHWs can download the entire program. One person can visit an internet-accessible location, download the modules, and bring these files back to share with others.
- **Reach**: The growing ownership and regular use of smartphones--even in remote regions--simplify delivery of the CHW training curriculum.
- **Bi-Lingual**: At present the CHW basic training curriculum is available in English and Spanish, and soon in French.

Online distribution allows the program to be accessible, immediate, and far-reaching, all key advantages for communities in remote and disproportionately affected regions.

### The learning environment

The training modules serve two complementary functions:

First, they help structure the classroom lessons taught by medical professionals who come from the region where the CHWs will serve. Using a monitor or projector to display the interactive material, the instructor leads the class through each module, engaging students in discussion, especially during the Q&A blocks throughout the module.^1^ In classroom presentations, instructors add information relevant to the local health environment. Using local dialects and references, they reflect on local health conditions, cultural norms, government requirements and resource availability. In this way, the instructors make the lessons culturally appropriate and relevant to each CHW team.

Second, the modules serve as valuable study guides for CHWs. The modules, installed on the learners’ phones or tablets, allow them to prepare for class and follow-up study after class. Even after course completion, the CHWs retain the modules for easy reference.

### Follow-up continuing medical education

As we described, local medical professionals bring the global program to the local level. Another essential approach to localizing the CHW training program is by way of a rigorous CME follow-up. Here’s how it works:

The NGO’s medical writers have developed an extensive collection of interactive modules to cover more than 400 health topics—infectious illnesses, chronic diseases, prevention measures, sanitation topics, parenting, family planning and much more—written specifically for CHWs. Available through a custom app, the modules are listed in an online library that can be searched alphabetically and by topic (using the National Library of Medicine typology). CHWs browse the library and download selected modules to their mobile devices. CHWs can study the material at their convenience then take the scored final exam at the end of each module. Adopting conventions used by most physician CME programs (19), this program requires CHWs to self-report their credits using an online tracking system. Each CHW is required to earn 50 credits per year.

Additionally, when needed, the NGO’s writing team is prepared to rapidly develop material that trains CHWs to respond appropriately to emerging health threats. Within the past decade notable examples include Ebola, Zika and COVID-19. A newly formed team of physicians, nurses, veterinarians and environmental health specialists are now developing a training package that addresses specific One Health related topics, including zoonotic diseases, vector spread and other environmental threats from climate change. So, the training program starts with a basic core curriculum, then, over time, continues to grow CHW knowledge that expands their capacity to serve changing community health needs.

The extensive variety of topics in the module library allows CHWs to choose and download the modules most relevant to the health conditions they confront in their communities. Heavy rains and flooding, for instance, raise the probability of cholera and other water-borne diseases. To prepare themselves and their communities, the CHWs can focus on relevant topics, such as cholera, typhoid fever, water purification procedures, diarrhea, oral rehydration therapy. The always-open CME library allows CHWs to learn about impending health issues or to refresh their understanding of pressing topics at any time.

The CME program allows CHWs to localize their skills and practices, but it also gives them an opportunity to “specialize” in their expertise. CHWs can choose packages of modules that expand their study for particular health topics. For instance, they can focus on maternal and child health, diabetes management, prevention of and living with HIV/AIDS. This specialized training equips CHWs with a more thorough understanding of common health conditions prevalent in their communities.

### Adoption of the program

The primary objective of this CHW training program is to empower small disproportionately affected communities to establish paraprofessional CHW teams. The aim is to lower the threshold of adoption—to ease access to a program that prepares their CHWs. These teams are equipped to deliver essential health services and to promote illness prevention strategies for local populations. Communities benefiting from this program are most likely to fall into the growing health equity gap. Thus, such a program will become even more essential over time.

What happens at the conclusion of training? First, local communities can contact the NGO if they want students to receive a certificate for completing the course. Learners will register for a comprehensive online exam that is monitored in real-time. Successful students (scoring at least 80%) receive a certificate of completion. Second, all new CHWs are enrolled in the CME program and provided with a CME app for their phones.

### Results to date

While this program has been implemented in several locations, we focused on one program where a well-established management team gathers weekly data on CHW outreach efforts. In Kisumu, Kenya, 20 CHWs are deployed 25 h/week in low-resource communities. Each CHW completes a report about the activities provided--clinical services or in prevention training sessions—health topics addressed and numbers of community members they served. These statistics are tabulated into monthly reports as an anecdotal measure of CHW effectiveness.

This CHW team received basic training early in 2020, completed 1 month before the COVID-19 pandemic struck. Well before its scheduled start date, the group of 20 CHWs was quickly pressed into service, and has been in service ever since. Initially, in public outdoor training sessions, they provided the only available source of COVID-19 prevention information, stressing mask wearing and rigorous hygiene measures. As the pandemic wore on, their activities expanded, providing clinical services, working with expectant mothers and offering a wide range of public training sessions covering dozens of topics in infectious and non-communicable diseases. As envisioned by the WHO, these CHWs also served as critical bridges to the over-stressed professional medical community, referring patients in need of higher-level care and providing follow-up care for people treated by a physician. For instance, these follow-ups include medications adherence, infection control and vital signs measurement and other monitoring as requested by the clinician.

In addition to these important services, especially during the pandemic, the CHW training program, in 2022, provided upgrade training in COVID-19 vaccine administration. In a classroom and clinical training setting (lasting 40 h), the health workers were instructed how to set up a shot clinic, according to WHO routines—from registration to post-vaccination observation. Vaccination training was a natural follow-up to the CHW core training. It demonstrates the value of having a prepared team of health workers available for quick response to a variety of unexpected health threats.

Verified monthly reports show that from late 2020 until the present day, the CHW team in Kisumu reaches more than 9,000 people each month with clinical services, health follow-ups and public health training sessions. In addition to the reported monthly activity reports, CHWs are invited to provide brief narratives describing the people they encounter and the health services they provide in their community. These accounts are collected, transcribed and cataloged as anecdotal evidence of the work CHWs accomplish on a routine basis. A sample of such narratives include:

1. **Health Screening Clinics**: “*HSCs are an innovative way to reach out to meet the needs of large numbers of the most vulnerable people in the community. The health screening clinics (clinical outreach) help improve the community’s awareness of health issues and inspire skill development among the CHWs. The clinic’s activity helps to improve the health outcomes. They lower the mortality rates because we encounter sick patients who are unable to go to hospital because of lack of health insurance. We can help them at the HSC or bring them in for higher-level care*.”—CHW.
2. **Teaching about an Emerging Disease: Mpox**: “*At the end of August I began educating members of my community about the deadly mpox virus, which has been confirmed in Kenya. I explained that the virus can spread from person to person through close contact with respiratory secretions and skin lesions. People thought mpox came only from monkeys. I explained transmission from animals to humans and then humans to humans. I educated them on prevention measures and advised them to report to the nearest health facility in case they developed any of the symptoms, especially any skin lesions*.”—CHW.
3. **Preventing Cholera through Habitual Water Treatment**: “*Many people in our community use untreated water thinking that is safe. However, along the way, before the water reaches consumers, pipes often burst, and the water mixes with dirty water and even sewage. This contamination is a reason that cholera is so rampant in our area. So, as a CHW in the field I teach my community members that they should treat water (which we explain) no matter where they draw it from to prevent them from getting waterborne diseases.*”—CHW.
4. **Persistence Pays Off for an HIV/AIDS Patient**: “*In my rounds, I came across a woman with HIV/AIDS symptoms who was in denial about having the disease. For three weeks, I talked to her about the benefits of taking antiviral drugs. Eventually she said to me that she would not take the drugs from any of the nearby hospitals, because she feared being labeled with HIV/AIDS. I took the woman to a more distant hospital for HIV/AIDS treatment and follow up with her to be sure she is taking her drugs regularly.*”*—*CHW.
5. **Educating My Community about Heart Disease**: “*heart disease happens only in people who are overweight,” is a commonly held belief in our community. In classes I teach participants about high blood pressure posing a big risk for heart disease. I teach people to take their blood pressure regularly (or I can take it for them) so that if it is high they can seek medical attention as soon as possible. I also advise residents about healthy diets and what foods can lead to high blood pressure and heart disease.*”—CHW.

## Discussion

Over the years, the WHO has strategically supported CHW programs to extend the reach of professional medical professionals and bridge the access gap to primary healthcare services. The training program described in this paper meets multiple criteria: it is cost-free, offers a professional, evidence-based curriculum developed by medical experts, it is downloaded to phones, accessible offline and adaptable to local health conditions and resources. While the core curriculum is comprehensive, ongoing CME ensures that CHWs maintain their basic knowledge and expand their skills. This program empowers small communities to harness the potential of CHWs in bridging the access gap and enhancing primary healthcare delivery in resource-limited settings.

It is important to stress the importance of the last point. While large, international organizations offer CHW training programs, these training efforts are often unavailable or unaffordable in small communities facing economic hardship, already lacking adequate medical care and disease prevention information. Thousands of communities fall into this health gap, and as the WHO reports, there is little expectation of resolving the gap with teams of doctors and nurses. Health conditions will only grow much worse as the forces of climate change grow stronger in the years ahead.

This CHW training program offers many solutions. It avoids most financial hurdles; it encourages local adoption and implementation without involving large bureaucratic institutions or lengthy application procedures. The evidence-based, professional curriculum properly prepares CHWs, and several provisions render the training relevant to local conditions. Finally, the all-important CME component, supported by non-profit sponsors, ensures that the CHWs continue their development as community health needs and conditions change over time.

In the authors’ view, this program is a viable and effective solution to the expanding global healthcare gap in low-resource communities.

## Data Availability

The original contributions presented in the study are included in the article/supplementary material, further inquiries can be directed to the corresponding author.

## Footnotes

^1^In regions entirely off the grid, the program provides solar-driven, low power projectors for classroom use.
